# Prevalence of depressive symptoms among nurses in China: A systematic review and meta-analysis

**DOI:** 10.1371/journal.pone.0235448

**Published:** 2020-07-07

**Authors:** Nanzhen Xie, Yan Qin, Taiwu Wang, Ying Zeng, Xia Deng, Li Guan

**Affiliations:** 1 Chongqing General Hospital, University of Chinese Academy of Sciences, Chongqing, People’s Republic of China; 2 Center for Disease Control and Prevention of Eastern Theater Command, Nanjing, People’s Republic of China; Gachon University Gil Medical Center, REPUBLIC OF KOREA

## Abstract

**Background:**

Depression is one of the most common mental disorders, profoundly impacting an individual’s performance and quality of life. Due to their unique working conditions, nursing is counted among the occupational groups at high risk for developing depression. Because of the shortage of nursing resources in China, Chinese nurses suffer from heavy daily workloads more than those in many other countries. Therefore, this study aimed to evaluate the overall prevalence of depressive symptoms and analyse the potential risk factors of depressive symptoms in Chinese nurses.

**Methods:**

A systematic literature search in PubMed, EMBASE, Web of Science, the Chinese BioMedical Literature Database (CBM), the China National Knowledge Infrastructure (CNKI), and the Weipu and Wanfang databases up to Dec 31st, 2019 was performed regarding the prevalence of depressive symptoms in Chinese nurses. Eligibility assessment and data extraction were performed independently by 2 researchers, and meta-analysis was used to synthesize the data. Heterogeneity was evaluated using Cochran’s Q test and quantified using the *I*^*2*^ statistic. To explore the potential source of heterogeneity, subgroup analyses were also performed. In addition, both funnel plot and Egger’s tests were adopted to assess publication bias.

**Results:**

A total of 102 studies published from 1996 to 2019 covering 22 provinces were included for further analysis. The total number of participants was 52,592, with a range of 46 to 7205 per study. The overall prevalence of depressive symptoms in Chinese nurses was 43.83% (95%CI: 40.26%-47.42%), and 31.12% (95%CI: 27.30%-35.07%) were classified as mild degrees of depressive symptoms. The prevalence of depressive symptoms may be significantly affected by region, province or municipality and department marital status. Moreover, an increasing trend in the prevalence of depressive symptoms was observed in recent years.

**Conclusion:**

The results presented a high prevalence of depressive symptoms among Chinese nurses, which suggests interventional programmes by health decision-makers to improving the mental state of nurses is needed urgently, especially in nurses with high risk factors for depressive symptoms. Furthermore, the nationwide investigation of depressive symptoms prevalence should be performed with a standard diagnostic tool, which may be more useful for policy makers and planners.

## Background

Depression is one of the most commonly diagnosed mental disorders or statuses, sometimes resulting in serious damage to the patient’s work ability [1, 2], performance [3, 4], interpersonal communications, physical health [5, 6], and quality of life [7]; some cases of depression may even result in the patient committing suicide [8]. According to the World Health Organization (WHO), approximately 300 million people of all ages suffer from depression worldwide, with an increase of more than 18% between 2005 and 2015. The global point, one-year and lifetime prevalence of depression are 12.9%, 7.2% and 10.8% respectively [9]. Depression is one of the biggest sources of disability and imposes a considerable economic burden on society [10]. In addition, more women are affected by depression than men [11].

It has been reported that doctors and nurses are one of the highest risk groups for developing depression [12]. Special working conditions, such as burnout [13, 14], high tension, overloaded clinical work, and occupational stress, seriously threaten the mental health of nurses. In addition, nurses often have to witness many different life events, such as disease, trauma, and even death, which imposes further physical and psychological effects on them. Because of the shortage of resources for nurses in China, Chinese nurses suffer from heavy daily workload more than those in any other country. The psychological status of nurses not only directly affects their own health but also affects the quality of medical care provided for their patients in a hospital setting [15]. Some studies have shown that the most common psychological problems experienced by nurses are anxiety and depression [16], and the incidence of depression in nurses has been showing an increasing trend [17, 18]. At present, relevant studies at home and abroad have found that there is a very high prevalence of depression in the nurse population [19, 20]. For example, studies from USA, Taiwan, and South Korea found the depressive symptoms prevalence in nurses population ranged from 18% to 61.7% [21–25]. Furthermore, a total of 46 cases of nurse suicide were reported or published from 2007 to 2016 [26]. Although various studies have been published in different regions in Chinese mainland, there has been no systematic comprehensive study about the prevalence of depressive symptoms. Therefore, the primary aim of this study is to quantitatively assess the prevalence of depressive symptoms in nurses from Chinese mainland and its primary related influencing factors by systematic review and meta-analysis.

## Methods

This study was performed based on the Preferred Reporting Items for Systematic Reviews and Meta-Analyses (PRISMA) [27]. To avoid potential biases from researchers, two authors (NX and YQ) conducted the study search and selection, quality assessment, and data extraction separately. The opinion of the third author (LG) was sought for and acted as a referee if any disagreement occurred or was otherwise necessary.

### Search strategy

All potential articles from PubMed, EMBASE, Web of Science, the Chinese BioMedical Literature Database (CBM), the China National Knowledge Infrastructure (CNKI), and the Weipu and Wanfang databases were obtained by electronic search. The last search for all databases was performed on Dec 31st, 2019. The keywords used for relevant studies were (“Prevalence” OR “Frequency” OR “Epidemiology”) AND (“Depression” OR “Mental Health Disorder” OR “Major Depression Disorder” OR “Mood disorder” OR “Affective disorder”) AND (“Nurses” OR “Nurse”) AND (“China” OR “Chinese”). Each keyword was searched individually or in combination to avoid missing relevant articles and maximize outputs.

### Inclusion and exclusion criteria

Manuscripts that fulfilled all the following criteria were included for further analysis: (1) cross-sectional study, or cohort studies that reported the prevalence of depressive symptoms; (2) targeted objects were nurses in Chinese mainland; (3) data available for depressive symptoms prevalence and corresponding depression scale; (4) the depression measuring scales adopted for depression assessment were well recognized internationally, for example, Zung's Self-Rating Depression Scale (SDS) [28].

Studies that met the following criteria were excluded: (1) not an original study, such as a review or editorial; (2) non–peer-reviewed local or government report or conference abstract; (3) studies from regions of China other than Chinese mainland (including Hong Kong, Macao, and Taiwan); (4) duplicate published studies; (5) nurses that were in specific training stages: students, standardized training or rotation; (6) nurses with specific characteristics, including pregnancy, perimenopause, and nurses suffering from trauma following an earthquake; (7) studies with small sample sizes (n<45).

### Quality assessment

To evaluate the selected articles, The ‘AHRQ Cross-Sectional/Prevalence Study Quality Checklist’ [29, 30] was used as a research instrument. which is the most widely accepted quality assessment tool for a cross-sectional study [31]. This instrument is available at http://www.ncbi.nlm.nih.gov/books/NBK35156/ and also can be found in the S1 Table. The instrument includes 11 items, which are answered with "yes", "no" and "unclear" respectively. 1 point will be given if one item is satisfied, and 0 point will be given for items not involved or unclear in the study. Article quality was assessed as follows: low quality = 0–3; moderate quality = 4–7; high quality = 8–11. The evaluation was conducted independently by two authors, and possible disagreements were settled through discussions with a third author.

### Data extraction

The following information was extracted from all included studies: title, year of publication, province, sample size, number of positive cases, diagnostic methods and other potential factors that may affect the prevalence of depressive symptoms in nurses and that was provided in the studies. Some of studies did not contain all the above-mentioned variables.

### Statistical analysis

Point estimates and 95% confidence intervals (95%CIs) for the prevalence rate of depressive symptoms in nurses were calculated for each study. To avoid having a confidence interval (CI) outside of the 0–1 range as well as studies with large weightings when the prevalence proportion becomes too small or too large, we calculated prevalence estimates with the variance-stabilizing double arcsine transformation [32]. Statistical heterogeneity was evaluated by Cochran’s Chi-squared test (with *P* < 0.10 indicating statistically significant heterogeneity) and the statistic *I*^2^ [33]. Heterogeneity with an *I*^2^ of 0 to 40% was treated as not important, while an *I*^2^ of 30 to 60% was treated as moderate heterogeneity, *I*^2^ of 50 to 90% was treated as substantial heterogeneity and *I*^2^ of 75 to 100% was treated as considerable heterogeneity [34]. If obvious heterogeneity existed (with *P* < 0.10), a random effects model was adopted for pooled results; otherwise, a fixed effects model was adopted. Fixed-effect models assume that the population effect sizes are the same for all studies [35]. In contrast, random-effects model attempted to generalize findings beyond the included studies by assuming that the selected studies are random samples from a larger population [36]. Furthermore, to identify potential influential studies, sensitivity analysis was performed by sequentially removing individual studies and evaluating the effect on the overall estimate. In addition, subgroup analysis was performed based on other potential sources of heterogeneity, such as province, regions (Northwest, Southwest, Northeast, South, Central, East and North China), severity of depressive symptoms, department, gender, age, job title, marriage, education background, shift work and hospital grade (if available). Furthermore, meta-regression was also performed to identify the causes of heterogeneity or examine the impact of moderator variables on study effect size of the prevalence. Publication bias was examined by funnel plots, and statistical significance was assessed by Egger’s test. In addition, for the meta-analysis, we assumed that the included studies were a random sample from each study population. Meta-analysis was carried out with the “meta” package version 4.11–0 [37] and graphical representation with ggplot2 package version 3.3.0 [38] of R language version 3.6.3 [39].

## Results

### Searching results and characteristics of the included studies

**Fig 1:**
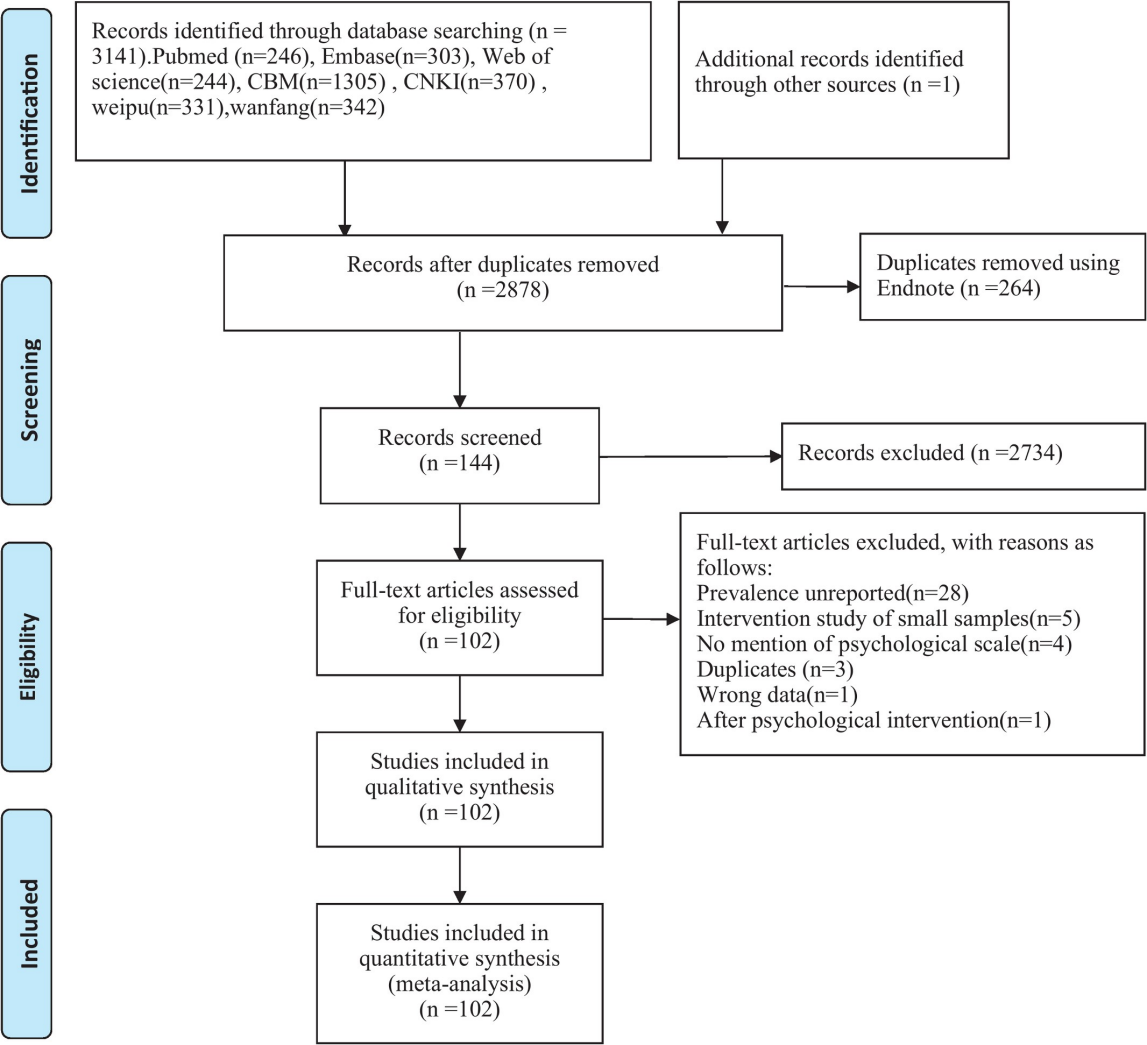
Flowchart describing the study design process.

Through the initial search, a total of 3142 potentially relevant citations were identified. A total of 264 duplicate papers were removed first, and 2734 papers were excluded after scanning their titles and abstracts. After screening the full texts of the included articles, 42 studies were excluded for the following reasons: Prevalence unreported(n = 28); Intervention study of small samples(n = 5); No mention of psychological scale(n = 4); Duplicates (n = 3); Wrong data(n = 1); After psychological intervention(n = 1). Finally, a total of 102 studies meeting the inclusion and exclusion criteria were included for further analysis (Fig 1), of which 3 were published in English-language journals, and the others were published in Chinese-language journals.

The basic characteristics of the final included studies are shown in S1 Table. These studies were published ranging from 1996 to 2019, covering 22 provinces, autonomous regions and municipalities. The scales used for depression assessment were listed as follows: Zung's Self-Rating Depression Scale (SDS) [28], Centre for Epidemiologic Studies-Depression Scale (CES-D) [40], Beck Depression Inventory (Beck) [41], Beck Depression Inventory (2nd ed) (BDI-II) [42], Hospital Anxiety and Depression Scale (HADS) [43], Hamilton Depression Rating Scale (HAMD) [44], and Patient Health Questionnaire (PHQ-9) [45]. The total number of participants was 52,592, with a range of 46 to 7205 per study.

### Quality evaluation

The AHRQ Cross-Sectional/ Prevalence Study Quality Checklist was applied to evaluate the study quality (S1 and S2 Tables). Among the selection items, the evaluation results ranged from 2 to 8, with the median score was 4. Overall, 65 of 102 studies have moderate or high quality, indicating a medium quality of the studies included.

### Overall prevalence of depressive symptoms in nurses from Chinese mainland

**Fig 2:**
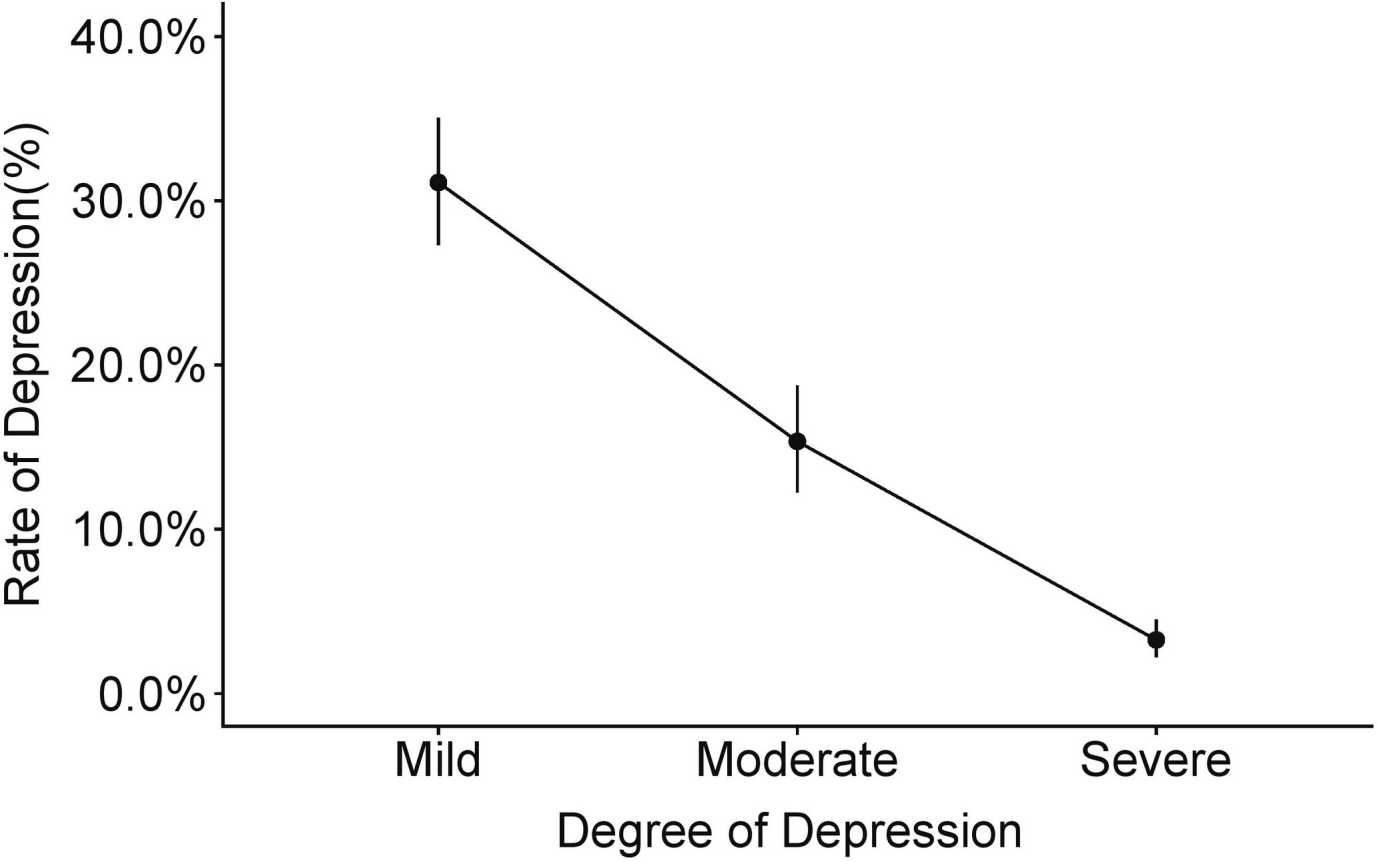
The prevalence of different degrees of depressive symptoms in Chinese nurses.

A total of 28,382 cases among the 52,592 nurses in the studies were found to have different degrees of depressive symptoms; the overall prevalence of depressive symptoms in nurses was 43.83% with 95%CIs of 40.26%-47.42%, with significant heterogeneity (*I*^*2*^ = 98.50%, *P* < 0.01). A total of 37 studies reported different degrees of depressive symptoms. After we pooled the results based on the severity of the depressive symptoms, the pooled prevalence and 95%CIs were 31.12% [27.30%; 35.07%] for mild depressive symptoms, 15.35% [12.24%; 18.74%] for moderate depressive symptoms and 3.26% [2.20%; 4.49%] for severe depressive symptoms, with *I*^*2*^ values of 92.30%, 96.00% and 96.50%, respectively. (Fig 2)

### Prevalence of depressive symptoms in nurses in relation to geographic regions and time

Geographic analysis based on provinces and regions was performed. We found that the highest prevalence of depressive symptoms was in nurses from the Northeast (54.69%, 95%CI: 50.51%-58.84%) and the lowest was in South China (37.59%, 95%CI: 32.75%-42.56%) (Table 1). The prevalence of depressive symptoms in nurses among different provinces is shown in Table 1. The highest and lowest prevalence of depressive symptoms were found in Hubei province (58.62%, 95%CI: 49.55%-67.41%) and in Inner Mongolia (22.41%, 95%CI: 5.39%-46.20%), respectively. Overall, the geographic location could significantly affect the prevalence, regardless of whether it was broken down by region or province.

**Table 1:** Comparison of prevalence rates in different regions/provinces of Chinese mainland.

Factor	Categories	No. of	No. of	No. of	Prevalence[95%CI]	Heterogeneity test	
		studies	participants	positive	(%)	*I*^*2*^(%)	*P*
Regions							
	South China	18	7291	2771	37.59[32.75;42.56]	93.00	*P* < 0.01
	Central China	15	17182	11674	48.08[39.26;56.97]	98.90	*P* < 0.01
	East China	27	6670	2796	41.40[35.07;47.87]	96.40	*P* < 0.01
	North China	19	7445	3798	43.49[34.36;52.84]	98.40	*P* < 0.01
	Northeast	12	9618	5444	54.69[50.51;58.84]	93.60	*P* < 0.01
	Northwest	5	1027	408	41.70[31.11;52.68]	91.80	*P* < 0.01
	Southwest	5	1886	715	40.75[33.43;48.28]	87.00	*P* < 0.01
Provinces							
	Anhui	1	231	111	48.05[41.62;54.51]	--	--
	Beijing	7	1516	716	42.23[26.51;58.80]	97.50	*P* < 0.01
	Fujian	1	632	253	40.03[36.24;43.88]	--	--
	Guangdong	18	7291	2771	37.59[32.75;42.56]	93.00	*P* < 0.01
	Hebei	6	3970	2491	54.78[46.12;63.29]	95.70	*P* < 0.01
	Henan	4	886	228	25.89[18.55;33.97]	85.10	*P* < 0.01
	Heilongjiang	2	587	304	51.96[44.84;59.05]	67.50	*P* < 0.01
	Hubei	5	1504	848	58.62[49.55;67.41]	91.10	*P* < 0.01
	Hunan	6	14792	10598	55.33[46.02;64.44]	98.70	*P* < 0.01
	Jilin	1	450	210	46.67[42.07;51.29]	--	--
	Jiangsu	6	736	218	33.05[15.56;53.32]	96.50	*P* < 0.01
	Jiangxi	1	77	36	46.75[35.67;57.99]	--	--
	Liaoning	9	8581	4930	56.14[51.40;60.82]	94.40	*P* < 0.01
	Inner mongolia	2	160	43	22.41[05.39;46.20]	88.90	*P* < 0.01
	Qinghai	1	163	90	55.21[47.51;62.79]	--	--
	Shandong	8	2147	878	40.68[30.59;51.18]	95.80	*P* < 0.01
	Shanxi	3	776	326	46.83[33.16;60.74]	92.70	*P* < 0.01
	Shaanxi	3	546	228	42.10[30.88;53.75]	85.60	*P* < 0.01
	Shanghai	2	183	86	44.18[17.87;72.36]	93.40	*P* < 0.01
	Sichuan	4	778	285	42.09[30.41;54.22]	89.90	*P* < 0.01
	Xinjiang	1	318	90	28.30[23.48;33.39]	--	--
	Zhejiang	8	2664	1214	46.35[33.40;59.55]	97.70	*P* < 0.01

Test for between-group differences of regions: Q = 31.89, *P* < 0.0001; Test for between-group differences of provinces: Q = 130.36, *P* < 0.0001.

**Fig 3:**
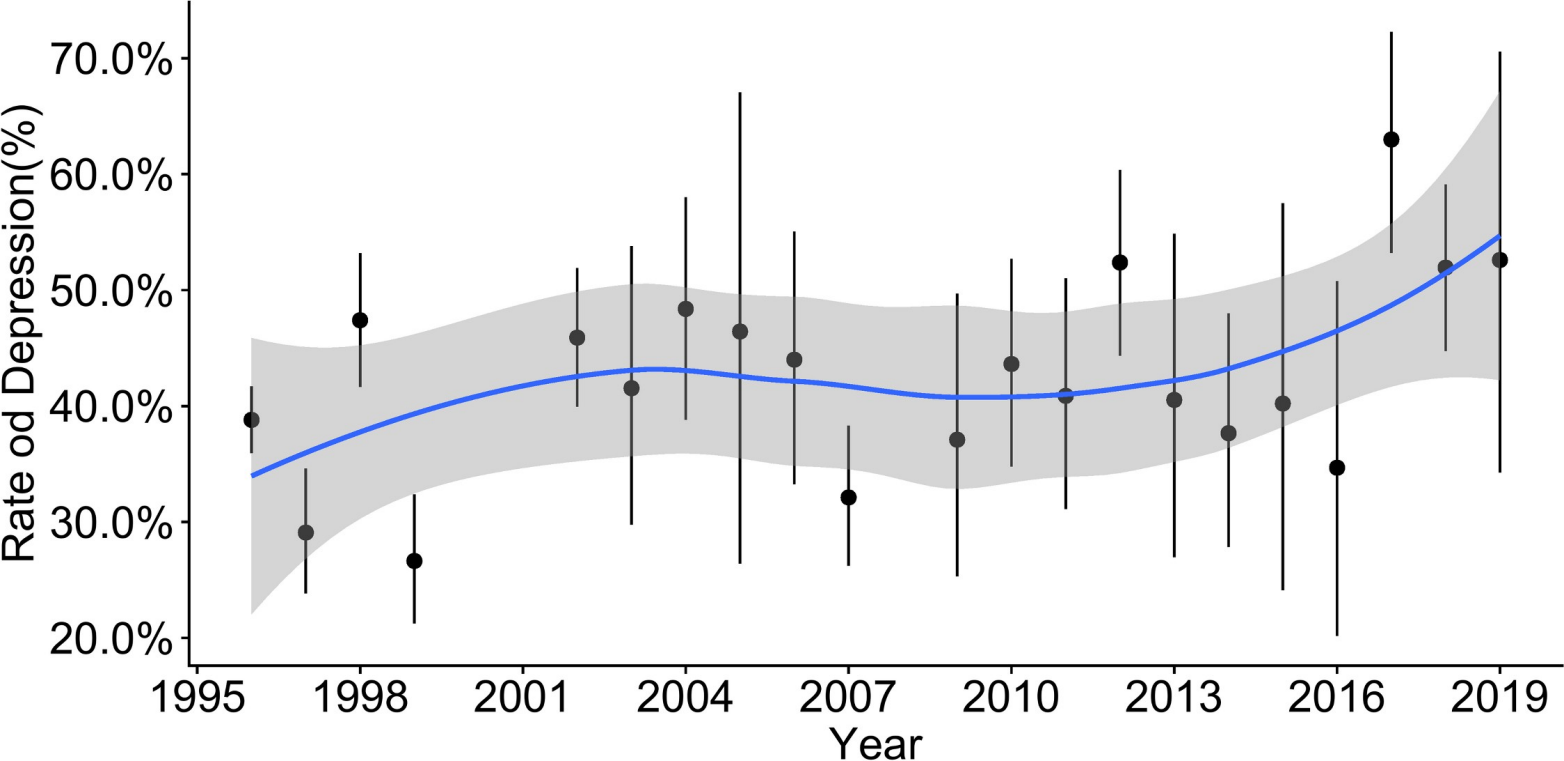
Time trend of depressive symptoms prevalence by year in Chinese nurses.

We also performed subgroup analysis by year. As shown in Fig 3, the lowest and highest prevalence were 26.64% (95%CI: 21.27%-32.38%) in 1999 and 62.99% (95%CI: 53.22%-72.26%) in 2017, respectively. Concerning the results of subgroup differences for prevalence in different years, a significant difference in terms of the prevalence trends was also found (*P* < 0.01).

### Prevalence of depressive symptoms in nurses in relation to risk factors

Other factors that may affect the prevalence of depressive symptoms in nurses were also analysed. The pooled estimates by potential risk factors associated with depressive symptoms in nurses are presented in Tables 2 and 3. Of all factors analysed in our study, the prevalence of depressive symptoms was significantly affected by department (Table 2); the lowest prevalence was in out-patient department, with 20.88% [16.03%, 26.15%], and the highest was in infectious diseases, with 58.21% [49.73, 66.45%]. In addition, we also found that marriage, educational background, age, job title, hospital grade, and shift work did not significantly affect the prevalence of depressive symptoms in nurses (Table 3).

**Table 2:** Comparison of prevalence in different departments of Chinese mainland.

Department	No. of	No. of	No. of	Prevalence [95%CI]	Heterogeneity test	
	studies	participants	positive	(%)	*I*^*2*^(%)	*P*
ICU	8	1666	758	45.34[36.82;53.99]	87.60%	*P* < 0.01
Infectious diseases	1	134	78	58.21[49.73;66.45]	--	*--*
Pediatrics	4	725	392	54.42[50.47;58.35]	27.80%	*P* = 0.25
O&G	7	893	389	41.62[27.76;56.15]	91.60%	*P* < 0.01
Emergency	9	720	334	35.51[22.29;49.87]	91.20%	*P* < 0.01
Psychiatry	14	2328	853	37.93[29.45;46.79]	94.70%	*P* < 0.01
Out-patient department	5	264	56	20.88[16.03;26.15]	0.00%	*P* = 0.74
Internal medicine	6	616	270	37.64[19.57;57.62]	95.50%	*P* < 0.01
Operation room	5	453	175	34.96[23.20;47.69]	85.30%	*P* < 0.01
Surgery	10	1556	654	32.79[22.63;43.81]	94.10%	*P* < 0.01
Hemodialysis room	3	183	84	45.80[38.39;53.30]	0.00%	*P* = 0.58
Oncology	3	2000	988	43.15[30.20;56.60]	95.80%	*P* < 0.01

*ICU: Intensive Care Unit; O&G: Obstetrics and Gynecology. Test for between-group differences: Q = 89.81, d.f. = 11, P < 0.0001.

**Table 3:** Prevalence of depressive symptoms in nurses associated with risk factors.

Factors	Categories	No. of studies	No. of participants	No. of depressive symptoms	Prevalence[95%CI] (%)	Heterogeneity		Between-group differences	
						*I*^*2*^(%)	P-value	Q	P-value
Age								2.18	0.34
	<30	18	4367	2120	44.89 [36.57; 53.34]	96.50%	*P* < 0.01		
	30–40	17	3251	1829	51.79[44.32; 59.22]	93.90%	*P* < 0.01		
	>40	18	2682	1352	45.06 [38.89; 51.31]	88.50%	*P* < 0.01		
Education level									
	Vocational School	14	2485	1313	51.53 [44.79; 58.25]	88.90%	*P* < 0.01	0.51	0.92
	Junior college	15	3824	2215	52.62 [46.54; 58.67]	91.80%	*P* < 0.01		
	Bachelor degree	12	2323	1318	53.72 [46.64; 60.74]	85.10%	*P* < 0.01		
	Graduate	2	410	208	50.75 [45.74; 55.74]	00.00%	*P* = 0.32		
Job title									
	Elementary	11	5057	1829	45.81[36.14;55.64]	97.50%	*P* < 0.01	0.10	0.75
	Intermediate or higher	11	1971	756	48.24[36.64;59.92]	95.10%	*P* < 0.01		
Marriage								2.16	0.34
	Single	9	2236	1270	55.71[50.87;60.50]	72.00%	*P* < 0.01		
	Married	9	4854	2782	55.58[50.87;60.23]	88.80%	*P* < 0.01		
	Divorce/Widowhood/Separation	2	105	74	85.22[45.15;100.00]	79.50%	*P* = 0.03		
Shift work									
	Yes	8	6407	3070	47.66 [37.45; 57.97]	98.4%	*P* < 0.01	0.65	0.42
	No	8	4694	2131	41.62 [31.47; 52.13]	98.0%	*P* < 0.01		
Gender*								0.85	0.36
	Male	6	265	148	55.97 [49.77; 62.08]	0.00%	*P* = 0.46		
	Female	6	1272	689	51.53 [44.71; 58.32]	81.20%	*P* = 0.03		
Hospital grade								0.11	0.94
	1	2	1608	880	46.09 [18.85; 74.68]	99.00%	*P* < 0.01		
	2	5	3494	1583	50.06 [39.63; 60.49]	96.20%	*P* < 0.01		
	3	6	4845	2751	51.49 [39.00; 63.89]	98.40%	*P* < 0.01		
Occupation								0.04	0.83
	Head nurse	2	496	271	61.43 [41.24; 79.79]	90.10%	*P* < 0.01		
	Nurse	2	1944	1129	59.17 [54.21; 64.04]	70.00%	*P* = 0.07		

*: Although most nurses in Chinese mainland are female, as most studies didn’t clearly state the proportion of female, we didn’t include these studies for subgroup analysis.

### Publication bias and sensitivity tests

**Fig 4:**
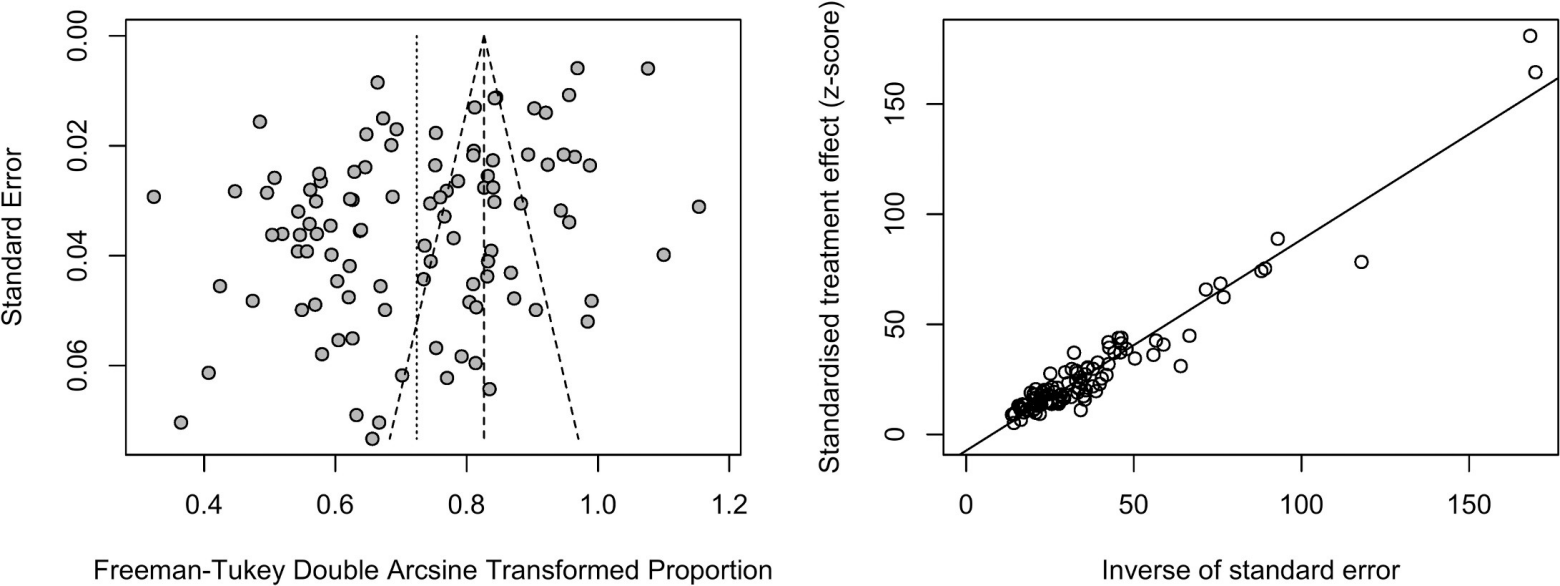
Funnel plot and Egger’s plot of depressive symptoms prevalence, showing potential publication bias.

Publication bias was examined by funnel plot and Egger’s test. A funnel plot shows that publication bias may exist (Fig 4), which was also confirmed by the result of Egger’s test (t = -6.20, *P* < 0.01). A sensitivity analysis for the pooled results was conducted by sequentially removing individual studies, and no significant differences before and after pooling were found, indicating stability in the pooled results.

## Discussion

Along with the rapid development of the economy in China, psychological problems have become increasingly more common. Nurses, as an important role in hospitals, have been increasingly demonstrating depressive symptoms. Although many articles have been published to assess the prevalence of depressive symptoms in Chinese nurses, a comprehensive study on this population is still absent. In our study, a total of 102 studies with 52,592 participants were obtained to assess the prevalence of depressive symptoms in mainland Chinese nurses. To our knowledge, this is the most comprehensive report to date to estimate that estimates this statistic, which may provide useful and valuable information for health decision-makers, helping them to properly implement interventional programmes and prevention activities.

In our study, the overall prevalence of depressive symptoms in mainland Chinese nurses was 43.83%, with 95%CI of 40.26%-47.42%, and obvious heterogeneity was demonstrated. We also found that the prevalence may be affected by regions/provinces, hospital department, and time, and may not be affected by educational background, age, job title, marriage, hospital grade, or shift work. Because of the high prevalence of depressive symptoms in Chinese nurses, which may result in large problems for society overall, we suggest that decision-makers should take actions to aid nurses in safeguarding their psychological wellbeing.

In a previous meta-analysis [46], the prevalence of depression in nursing students worldwide was 34.0% and was affected by age and geographical regions, with Asian nursing students experiencing a higher prevalence (43.0%). We may see that both student and professional nurses, especially in Asia, have a very high prevalence of depression; this prevalence is higher than even that of older patients with diseases such as stroke, hypertension, diabetes and coronary heart disease [47], and is similar to that of empty-nest elderly individuals [48]. In addition, the prevalence of depression among Chinese nurses is higher than that of nurses in Iran [49] and Chinese Hong Kong [50, 51], Australian midwives [52], and Hungarian [53] and Australian [54] nurses. However, to our surprise, only 13.2% of nurses in Vietnam have depression [55], as well as 24.9% of Iranian nurses working in military hospitals [56]. In addition, the Chinese nurses even seem to have higher prevalence of depression than some special populations, such as people living with HIV with 38% [57], outpatients with 27.0% [58], and Indian elderly population with 34.4% [59]. Therefore, we may conclude that Chinese nurses were at a particularly high risk of having depressive symptoms. Moreover, based on the time trend shown in Fig 3, the prevalence of depressive symptoms among Chinese nurses may have increased in recent years, especially in large hospitals with a low ratio of doctors to nurses and of nurses to patients.

We also found that the prevalence of depressive symptoms was significantly different based on geographic distribution and hospital department. In total, we could see that nurses from the Hubei province and the Northeast region had the highest prevalence of depressive symptoms. This may be because of the occupational environment and policies in each region. From Table 2, we can see that the departments with the highest prevalence of depressive symptoms for nurses are infectious diseases, paediatrics, haemodialysis, ICU, and oncology. This may be due to the heavy workload and time pressures inherent in working in these departments. In addition, we also found that, in terms of marital status, despite no significant difference was found, divorce/widowhood/separation had higher prevalence than the others, which may be due to the sample size. To our surprise, we found that the prevalence of depressive symptoms in department of psychiatry wasn’t that high as we expect, which may be due to as follows [60]: 1) more professional education about mental health was obtained, 2) the workload and difficulty of nurses in department of psychiatry were easier than others, 3) as closed-off management was adopted in most department of psychiatry, they didn’t face the trouble from family members of patients, 4) psychopaths often didn’t have physical disease, 5) more medical disputes existed in general hospitals than psychiatric hospitals.

In China, there is a large shortage of resources for nurses, the ratio of the nurse population to the total population is 1:1750, which is much lower than that of some developed countries (1:140–1:320) [61]. Nurses are faced with heavy workloads, especially in the grade 3A hospitals in the city. However, the present situation cannot be changed in a short period of time. Due to the recent COVID-19 pandemic, the impact on mental health on healthcare workers is tremendous and more nurses suffer from depression [62, 63]. It is suggested that hospital managers should pay attention to the physical and mental state of their nurses, establish mechanism for the prevention and control of negative emotions such as depressive symptoms, formulate feasible measures to reasonably reduce the workloads of nurses, improve the working environment and the sense of occupational identity, improve and maintain the quality of life while ensuring the quality of medical service, and ensure the physical and mental health of the nurses. These steps may play a role in saving resources and improving nurses’ quality of life and work efficiency [64].

### Limitations

The strengths of this review include a comprehensive analysis of the literature to identify all potential articles related to the topic, a robust methodology in conducting the systematic review, and combining estimates generated from the meta-analyses. The meta-analysis results also have some limitations that should be acknowledged: 1) All studies used a cross-sectional observational study design; 2) Most of the literature included in this study was published in Chinese-language journals, with very few in English-language journals, the overall quality of included studies; 3) The criteria and cut-off for diagnosis varied with studies, which may have led to the heterogeneity observed; 4) Only 22 provinces in Chinese mainland have been covered with regards to the prevalence of depressive symptoms in their nurses, which may have led to deficiencies or inaccuracies in estimating the overall prevalence; 5) Some potential confounding factors were analysed to try and understand the high heterogeneity, but the main reason is still unknown; 6) As the limitation of sample size in some groups, such as department of infectious diseases, Anhui and Jilin provinces, some results still need further confirmations; and 7) Publication bias could not be avoided.

## Conclusions

Despite the considerably high heterogeneity and existence of publication bias in the study, the prevalence of depressive symptoms among nurses in Chinese mainland is higher than that in many other countries. As the worldwide prevalence of depression is expected to increase over the next few decades [65], these results could provide useful and valuable information for health decision-makers. Furthermore, the nationwide investigation of depression prevalence should be performed with a standard diagnostic tool, which may be more useful for policy makers and planners.

## Supporting information

S1 Table Baseline characteristics of the included studies. (DOCX)

S2 Table Quality evaluation of the 102 studies included in the meta-analysis. (DOCX)

S3 Table ARHQ methodology checklist for cross-sectional/ prevalence study. (DOC)

S1 Checklist PRISMA checklist. (DOC)

S1 Appendix The detailed information of search strategy. (DOC)
