# Supplementary material for: Prevalence of depressive symptoms among nurses in China: A systematic review and meta-analysis
Source: PLoS One. 2020 Jul 7;15(7):e0235448. doi: 10.1371/journal.pone.0235448 (PMC7340293; doi:10.1371/journal.pone.0235448)
Supplement: S1 Table — (DOCX) [file pone.0235448.s001.docx]

Table S1. Baseline characteristics of the included studies

| Author | Year | Region | Province | Method and cut -off score to assess depressive symptoms | Age (mean ± sd or min - max) | Department | Sample | Positive | Type of publication | Score of Quality |
| --- | --- | --- | --- | --- | --- | --- | --- | --- | --- | --- |
| Li et al.[[1](#_ENREF_1)] | 2005 | South China | Guangdong | SDS, 50 | - | ICU | 92 | 64 | Journal Article | 5 |
| Huang et al.[[2](#_ENREF_2)] | 2004 | South China | Guangdong | SDS, 50 | 30.87±8.18 | - | 779 | 283 | Journal Article | 3 |
| Huang et al.[[3](#_ENREF_3)] | 2004 | South China | Guangdong | SDS, 50 | 29.01±8.58 for Cancer hospital;  29.49±5.66 for Polyclinic | - | 408 | 141 | Journal Article | 3 |
| Hong et al.[[4](#_ENREF_4)] | 2004 | South China | Guangdong | SDS, 50 | 21-53 | Operation room;  Surgery | 142 | 48 | Journal Article | 2 |
| Xie et al.[[5](#_ENREF_5)] | 2009 | South China | Guangdong | SDS, 50 | 20-48 | - | 327 | 177 | Journal Article | 5 |
| Ling et al.[[6](#_ENREF_6)] | 2006 | Central China | Hunan | SDS, 50 | 31.0±8.88 | Operation room | 81 | 26 | Journal Article | 2 |
| Chen et al.[[7](#_ENREF_7)] | 2012 | Central China | Hubei | SDS, 50 | 31.4±8.1 | - | 384 | 210 | Journal Article | 5 |
| Mo.[[8](#_ENREF_8)] | 2006 | Central China | Hunan | SDS, 50 | 22-45 | Operation room | 157 | 49 | Journal Article | 4 |
| Shen et al.[[9](#_ENREF_9)] | 2012 | East China | Jiangsu | SDS, 50 | 29.17±6.01 | Infectious diseases | 134 | 78 | Journal Article | 6 |
| Lu et al.[[10](#_ENREF_10)] | 2015 | East China | Shandong | SDS, 50 | 32.77±7.07 for Mental hospital;  31.94±6.86 for Polyclinic | - | 356 | 106 | Journal Article | 2 |
| Yan.[[11](#_ENREF_11)] | 2011 | East China | Shanghai | SDS, 50 | 20-58 | Emergency department;  Out-patient department | 74 | 22 | Journal Article | 2 |
| Wang et al.[[12](#_ENREF_12)] | 2005 | East China | Shandong | SDS, 50 | 31.87±9.18 | - | 437 | 158 | Journal Article | 3 |
| Miao et al.[[13](#_ENREF_13)] | 1997 | East China | Shandong | SDS, 50 | 30.74±9.21 | - | 275 | 80 | Journal Article | 3 |
| Ye et al.[[14](#_ENREF_14)] | 2014 | East China | Zhejiang | SDS, 50 | 31.7±8.3 | Emergency department | 357 | 179 | Conference Article | 5 |
| Zhang et al.[[15](#_ENREF_15)] | 2005 | North China | Beijing | SDS, 50 | 28±5.8  26±5.6 | Obstetrics; Surgery | 82 | 28 | Journal Article | 2 |
| Wang et al.[[16](#_ENREF_16)] | 2007 | North China | Shanxi | SDS, 50 | 29.01 ±8.85 for Psychiatry;  29.49 ±5.66 for on-psychiatry | Psychiatry department;  Non-psychiatry department | 408 | 141 | Journal Article | 2 |
| Liu et al.[[17](#_ENREF_17)] | 2009 | North China | Beijing | SDS, 50 | 18-54 | - | 306 | 69 | Journal Article | 5 |
| wang.[[18](#_ENREF_18)] | 2015 | North China | Hubei, Beijing | SDS, 50 | 20-57 | - | 1023 | 222 | Thesis | 6 |
| Ren et al.[[19](#_ENREF_19)] | 2011 | Northeast | Liaoning | SDS, 50 | 20-35 | - | 198 | 70 | Journal Article | 4 |
| Cheng et al.[[20](#_ENREF_20)] | 2004 | Northeast | Liaoning | SDS, 50 | - | - | 247 | 162 | Journal Article | 3 |
| Yuan et al.[[21](#_ENREF_21)] | 2007 | Northwest | Shaanxi | SDS, 50 | - | - | 291 | 117 | Journal Article | 4 |
| Wang et al.[[22](#_ENREF_22)] | 1996 | Southwest | Sichuan, Yunnan, Guizhou, Xizang | SDS, 50 | - | - | 1108 | 430 | Journal Article | 3 |
| Tu et al.[[23](#_ENREF_23)] | 2013 | Southwest | Sichuan | SDS, 50 | 18-44 | - | 60 | 33 | Journal Article | 3 |
| Huang et al.[[24](#_ENREF_24)] | 2013 | Southwest | Sichuan | SDS, 50 | 32.51±8.58 for Nephrological;  32.62±8.53 for Hemodialysis room | Nephrological; Hemodialysis room | 200 | 71 | Journal Article | 3 |
| Zhu et al.[[25](#_ENREF_25)] | 2009 | North China | Inner Mongolia | SDS, 50 | 21- 45 | Obstetrics;  General surgery | 110 | 37 | Journal Article | 2 |
| Qu.[[26](#_ENREF_26)] | 1999 | East China | Shandong | SDS, 50 | 32.14±8.27 | - | 244 | 65 | Journal Article | 3 |
| Hong et al.[[27](#_ENREF_27)] | 2003 | South China | Guangdong | SDS, 50 | 33.14±8.8 | Operating Room | 65 | 27 | Journal Article | 3 |
| Huang et al.[[28](#_ENREF_28)] | 2004 | South China | Guangdong | SDS, 50 | 29.01±8.58 | Oncology departments | 213 | 60 | Journal Article | 5 |
| Xiang et al.[[29](#_ENREF_29)] | 2016 | South China | Guangdong | SDS, 53 | 34.23±5.81 | Psychiatry department | 120 | 20 | Journal Article | 3 |
| Zhang et al.[[30](#_ENREF_30)] | 2009 | South China | Guangdong | SDS, 53 | ＜35 | Emergency department;  ICU, et al | 190 | 51 | Journal Article | 3 |
| Deng et al.[[31](#_ENREF_31)] | 2011 | South China | Guangdong | SDS, 53 | 20-51 | Psychiatry department | 192 | 56 | Journal Article | 5 |
| Lin et al.[[32](#_ENREF_32)] | 2010 | South China | Guangdong | SDS, 53 | 31.5±2.1 | Emergency department;  Non-emergency department | 162 | 43 | Journal Article | 4 |
| Gong et al.[[33](#_ENREF_33)] | 2014 | South China | Guangdong | SDS, 53 | 31.93±7.55 | - | 3474 | 1320 | Journal Article | 6 |
| Zhu [[34](#_ENREF_34)] | 2011 | South China | Guangdong | SDS, 53 | 21-42 | NICU | 46 | 17 | Journal Article | 2 |
| Pan et al.[[35](#_ENREF_35)] | 2013 | South China | Guangdong | SDS, 53 | - | Psychiatry department | 192 | 47 | Journal Article | 5 |
| Li et al.[[36](#_ENREF_36)] | 2016 | East China | Anhui | SDS, 53 | - | - | 231 | 111 | Journal Article | 6 |
| Zhong et al.[[37](#_ENREF_37)] | 2013 | East China | Jiangsu | SDS, 53 | - | Out-patient department;  Emergency department | 66 | 10 | Journal Article | 5 |
| Gu.[[38](#_ENREF_38)] | 2006 | East China | Shanghai | SDS, 53 | - | Psychiatry department | 109 | 64 | Journal Article | 5 |
| Wang et al.[[39](#_ENREF_39)] | 2009 | East China | Zhejiang | SDS, 53 | 27.6±5.1 | NICU | 50 | 19 | Journal Article | 4 |
| Zheng et al.[[40](#_ENREF_40)] | 2018 | East China | Fujian | SDS, 53 | 31.34±7.9 | - | 632 | 253 | Journal Article | 7 |
| Jia et al.[[41](#_ENREF_41)] | 2015 | Central China | Henan | SDS, 53 | 31.58±16.47 | - | 280 | 96 | Journal Article | 7 |
| Chen.[[42](#_ENREF_42)] | 2014 | Central China | Henan | SDS, 53 | 22.12±2.10 | - | 312 | 58 | Journal Article | 6 |
| Song.[[43](#_ENREF_43)] | 2012 | Central China | Hubei | SDS, 53 | 26.45±7.32 | - | 64 | 31 | Journal Article | 5 |
| Luo et al.[[44](#_ENREF_44)] | 2011 | Central China | Hunan | SDS, 53 | - | - | 171 | 77 | Journal Article | 3 |
| Liu.[[45](#_ENREF_45)] | 2010 | Northwest | Qinghai | SDS, 53 | - | - | 163 | 90 | Journal Article | 3 |
| Yan et al. [[46](#_ENREF_46)] | 2004 | Northwest | Shaanxi | SDS, 53 | - | Operation room | 130 | 71 | Journal Article | 3 |
| Hui.[[47](#_ENREF_47)] | 2016 | Northeast | Liaoning | SDS, 53 | 31.29±6.25 | - | 800 | 374 | Thesis | 8 |
| Fang et al.[[48](#_ENREF_48)] | 2018 | Northeast | Heilongjiang | SDS, 53 | 35.01±9.33 | Otorhinolaryngology | 273 | 152 | Journal Article | 4 |
| Liu et al.[[49](#_ENREF_49)] | 2006 | North China | Hebei | SDS, 53 | - | Psychiatry department | 148 | 81 | Journal Article | 3 |
| Shi et al.[[50](#_ENREF_50)] | 2015 | North China | Hebei | SDS, 53 | 33.4±7 | - | 2158 | 1440 | Journal Article | 3 |
| Wei et al.[[51](#_ENREF_51)] | 2007 | Northwest | Shaanxi | SDS, 53 | - | - | 125 | 40 | Journal Article | 4 |
| Wang.[[52](#_ENREF_52)] | 2015 | Central China | Henan | SDS, 53 | 29.88±7.5 | - | 104 | 30 | Thesis | 6 |
| Liu et al.[[53](#_ENREF_53)] | 2018 | North China, East China | Beijing, Tianjin, Zhejiang, Shandong | SDS, 53 | 30.3±8.2 | Oncology departments | 1473 | 776 | Journal Article | 6 |
| Sun et al.[[54](#_ENREF_54)] | 2019 | Northwest | Xinjiang | SDS, 53 | 28.59±4.98 | - | 318 | 90 | Journal Article | 6 |
| Wang et al.[[55](#_ENREF_55)] | 2018 | North China | Beijing | SDS, Depression index≥0.5 | 33.7±7.4 | - | 268 | 160 | Journal Article | 4 |
| Cheng et al.[[56](#_ENREF_56)] | 2002 | North China | Shanxi | SDS, Depression index≥0.5 | - | - | 268 | 123 | Journal Article | 3 |
| Jiang et al.[[57](#_ENREF_57)] | 2013 | North China | Beijing | SDS, Depression index≥0.5 | 26.65±5.21 | - | 449 | 313 | Journal Article | 7 |
| Huang et al.[[58](#_ENREF_58)] | 2015 | Northeast | Jilin | SDS, Depression index≥0.5 | 32.12±1.86 | - | 450 | 210 | Journal Article | 3 |
| Liu et al.[[59](#_ENREF_59)] | 2014 | Northeast | Heilongjiang | SDS, Depression index≥0.5 | 28.76±6.15 | Oncology departments | 314 | 152 | Journal Article | 6 |
| Liu et al.[[60](#_ENREF_60)] | 2012 | Central China | Hunan | SDS, Depression index≥0.5 | - | - | 7071 | 5477 | Journal Article | 5 |
| Ouyang.[[61](#_ENREF_61)] | 2012 | Central China | Hunan | SDS, Depression index≥0.5 | - | - | 7205 | 4894 | Thesis | 8 |
| Gong et al.[[62](#_ENREF_62)] | 2012 | South China | Guangdong | SDS, Depression index≥0.5 | 36.5±7.6 | Psychiatry department | 100 | 39 | Journal Article | 3 |
| Han et al.[[63](#_ENREF_63)] | 2013 | South China | Guangdong | SDS, Depression index≥0.5 | - | - | 283 | 96 | Journal Article | 2 |
| Liang.[[64](#_ENREF_64)] | 1998 | South China | Guangdong | SDS, Depression index≥0.5 | - | Psychiatry department | 289 | 137 | Journal Article | 4 |
| Zhao.[[65](#_ENREF_65)] | 2007 | East China | Zhejiang | SDS, Depression index≥0.5 | 18-55 | - | 374 | 88 | Journal Article | 4 |
| Xu.[[66](#_ENREF_66)] | 2011 | East China | Shandong | SDS, Depression index≥0.5 | 38.7±8.8 | Psychiatry department | 106 | 55 | Journal Article | 3 |
| Xu et al.[[67](#_ENREF_67)] | 2010 | East China | Shandong | SDS, Depression index≥0.5 | 38.9±9.2 | Psychiatry department | 127 | 57 | Journal Article | 3 |
| Zhou et al.[[68](#_ENREF_68)] | 2004 | East China | Shandong | SDS, Depression index≥0.5 | 34.5±13 | Psychiatry department;  Non-psychiatry department | 148 | 68 | Journal Article | 5 |
| Mo et al.[[69](#_ENREF_69)] | 2019 | East China | Zhejiang | SDS, Depression index≥0.5 | - | - | 258 | 216 | Journal Article | 5 |
| Chen et al.[[70](#_ENREF_70)] | 2013 | Central China | Hubei | SDS, Total rough score≥40 | 31.1±7.9 | - | 571 | 300 | Journal Article | 4 |
| Ding et al.[[71](#_ENREF_71)] | 2007 | North China | Beijing | SDS, Total rough score＞40 | 28.6±5.36 | - | 209 | 65 | Journal Article | 5 |
| Wang et al.[[72](#_ENREF_72)] | 2017 | Southwest | Sichuan | SDS, Total rough score≥41 | 30.7±9.2 | - | 122 | 64 | Journal Article | 5 |
| Zhou et al.[[73](#_ENREF_73)] | 2006 | Southwest | Sichuan | SDS, Total rough score＞40 | 19-26 | - | 396 | 117 | Journal Article | 4 |
| Zhang et al.[[74](#_ENREF_74)] | 2006 | East China | Zhejiang | SDS, Total rough score≥41 | 28.2±7.35 | - | 162 | 45 | Journal Article | 4 |
| Li.[[75](#_ENREF_75)] | 2016 | North China | Hebei | SDS (No standard points mentioned) | 33.25±9.24 | Pediatrics | 487 | 270 | Journal Article | 6 |
| Zhang et al.[[76](#_ENREF_76)] | 2004 | North China | Beijing | SDS (No standard points mentioned) | 28.88 ±5.95 | ICU | 102 | 54 | Journal Article | 3 |
| Yu et al.[[77](#_ENREF_77)] | 2010 | Central China | Henan | SDS (No standard points mentioned) | 31.89±8.24 | - | 190 | 44 | Journal Article | 4 |
| Jiao et al.[[78](#_ENREF_78)] | 2006 | South China | Guangdong | SDS (No standard points mentioned) | 20-54 | Obstetrics | 217 | 145 | Journal Article | 2 |
| Geng.[[79](#_ENREF_79)] | 2012 | North China | Beijing | SDS (No standard points mentioned) | 28.43 ± 5.32 | - | 100 | 27 | Journal Article | 5 |
| Gao et al.[[80](#_ENREF_80)] | 2011 | Northeast | Liaoning | CES-D≥16 | 19-60 | - | 1277 | 809 | Journal Article | 4 |
| Gao et al.[[81](#_ENREF_81)] | 2011 | Northeast | Liaoning | CES-D≥16 | 35.01 ± 9.33 | - | 1437 | 886 | Journal Article | 5 |
| Zhao et al.[[82](#_ENREF_82)] | 2012 | Northeast | Liaoning | CES-D≥16 | 33.4±7.5 | - | 515 | 348 | Journal Article | 5 |
| Wu et al. [[83](#_ENREF_83)] | 2010 | Northeast | Liaoning | CES-D≥16 | 34.5 ±9.6 | - | 1986 | 1111 | Journal Article | 5 |
| Wu et al. [[84](#_ENREF_84)] | 2010 | Northeast | Liaoning | CES-D≥16 | 34.8 ± 9.8 | - | 1937 | 1079 | Journal Article | 5 |
| Wang et al.[[85](#_ENREF_85)] | 2012 | North China | Hebei | CES-D≥16 | 45-55 | - | 107 | 22 | Journal Article | 4 |
| Yuan et al.[[86](#_ENREF_86)] | 2017 | North China | Hebei | CES-D≥16 | 32.3±6.8 | - | 535 | 353 | Journal Article | 5 |
| Li et al.[[87](#_ENREF_87)] | 2006 | East China | Zhejiang | CES-D＞16 | 27±11 | - | 528 | 277 | Journal Article | 4 |
| Wan et al.[[88](#_ENREF_88)] | 2019 | East China | Jiangxi | CES-D≥16 | 18-45 | Internal medicine;  Surgery;  Obstetrics and Gynecology;  Pediatrics;  Hemodialysis room | 77 | 36 | Journal Article | 7 |
| Yan et al.[[89](#_ENREF_89)] | 2019 | North China | Hebei | CES-D≥16 | 20~65 | - | 535 | 325 | Journal Article | 5 |
| Huang et al.[[90](#_ENREF_90)] | 2009 | Northeast | Liaoning | Beck≥5 | 25-49 | - | 184 | 91 | Journal Article | 2 |
| Zhang et al.[[91](#_ENREF_91)] | 2017 | North China | Shanxi | Beck≥5 | - | ICU;Surgery;  Internal medicine;  Obstetrics and Gynecology | 100 | 62 | Journal Article | 5 |
| Li et al.[[92](#_ENREF_92)] | 2004 | East China | Shandong | Beck≥5 | 20-45 | - | 454 | 289 | Journal Article | 5 |
| Dai.[[93](#_ENREF_93)] | 2014 | East China | Jiangsu | Beck≥5 | - | - | 52 | 18 | Journal Article | 4 |
| Zhou.[[94](#_ENREF_94)] | 2004 | Central China | Hunan | Beck≥5 | - | Emergency department | 107 | 75 | Journal Article | 3 |
| Li et al.[[95](#_ENREF_95)] | 2016 | East China | Jiangsu | BDI-Ⅱ≥14 | 28.9±4.8 for day shift nurses; 27.4±4.7 for night shift nurses | Psychiatry department | 120 | 46 | Journal Article | 4 |
| Li et al.[[96](#_ENREF_96)] | 2016 | East China | Jiangsu | HADS≥8, | - | Psychiatry department | 291 | 29 | Journal Article | 4 |
| Wang et al.[[97](#_ENREF_97)] | 2018 | East China | Zhejiang | HAMD＞7 | 50-57 | - | 70 | 37 | Journal Article | 5 |
| Zhang et al.[[98](#_ENREF_98)] | 2017 | East China | Jiangsu | HAMD-17≥8 | 34.7±13.4 | Hemodialysis room | 73 | 37 | Journal Article | 6 |
| Ke et al.[[99](#_ENREF_99)] | 2015 | Central China | Hubei | CES-D≥16 | 38.5±6.7 | ICU | 328 | 182 | Journal Article | 3 |
| He et al.[[100](#_ENREF_100)] | 2017 | Central China | Hubei | PHQ-9≥5 | - | - | 157 | 125 | Journal Article | 5 |
| Wang.[[101](#_ENREF_101)] | 2011 | North China | Inner Mongolia | HRDS≥8 | 32.4±8.7 | Eemergency department | 50 | 6 | Journal Article | 4 |
| Dai.[[102](#_ENREF_102)] | 2019 | East China | Zhejiang | HADS＞7 | 32.49±10.35 for day shift nurses; 28.33±5.76 for night shift nurses | - | 865 | 353 | Journal Article | 6 |

-Unspecified age or department

**1.** Li Y, Wang X, Zhao H. Investigation and analysis of anxiety and depression and related factors in ICU nurses. Modern Clinical Nursing. 2005; 4(3): 6-8.

**2.** Huang H, Ying W, Xiao H, Wang H, Wang M. Analysis on occupational stress and mental health status of nurses. Chinese Nursing Research. 2004; 18(4A): 590-591.

**3.** Huang H, Xian M, HuiyingTan, Ying W, Wang M, Wang Y, et al. Mood Status of Nurses and Patients in Tumor Hospital and General Hospital. Chinese Mental Health Journal. 2004; 18(6): 387-389.

**4.** Hong R, Huang H, Ying W, Xiao H, Wang M, Wang Y. Comparison of Psychological Health Status Between Nurses at Operating Room and Nurses at General Surgery Ward. Chinese Journal of Practical Nursing. 2004; 20(236): 3-4.

**5.** Xie Z, Luo Y, Yao C, Luo W. An investigation on working pressure sourcea of clinical first-line nursing staff. International Medicine and Health Guidance 2009; 15(16): 13-15.

**6.** Ling S, Guo X. Investigation and analysis of mental health status of nurses in operating room. Today Nurse. 2006; (05): 92-94.

**7.** Chen C, Zhong B, Hua Y, Nie F, Wang X. Correlation between Depressive Symptom and Job Stress among Nurses from First-Class Hospitals in Wuhan. Journal of Environmental and Occupational Medicine. 2012; 29(8): 488-493.

**8.** Mo W. Analysis on the causes and correlation of fatigue syndrome of nurses in operating room. Today Nurse. 2006; (2): 22-23.

**9.** Shen J, Qian X, Gu P, Yang J, Sun Y, Zhang L, et al. A survey of depression status of nurses in department of infectious diseases. Chinese Nursing Research. 2012; 26(20): 1838-1839.

**10.** Lu Q, Zhong G. Depression in Psychiatric Nurses. China Journal of Health Psychology. 2015; 23(2): 204-206.

**11.** Yan X. Analysis on the causes of depression in emergency nurses and its prevention and treatment measures. Chinese And Foreign Women Health. 2011; 19(7): 278.

**12.** Wang C, Duan G, Li Y, Wang X, Liang L. Analysis of occupational stress and psychological factors of nurses. Foreign Medical Sciences(Nursing Foreing Medical Science). 2005; 24(4): 185-186.

**13.** Miao C, Xu L, Liu X, Li C, Zhou G, Chu L. Study on Depressive Symptoms and Related Factors of Nurses. Shangdong Archives of Psychiatry. 1997; (02): 23-25.

**14.** Ye M, Chen H, Lu Y, Chen Y, Cao Q, Chen X, et al., editors. Coexisting of depression and anxiety symptoms and its association with job stress among female nurses from emergency departments of general hospitals. Seventh National Conference on Mental Health 2014; Beijing China.

**15.** Zhang L, Yin J, Xue G. Comparison and Analysis on Psychological Health Status of Nurses in Delivery Room and in Surgery Ward. Modern Nurse. 2005; 11(16): 1291-1292.

**16.** Wang Y, Wang Z, Xia X, Zhao T. Comparative Analysis of emotional State and Occupational stress sources of nurses in Psychiatric Hospitals. Medical Journal of Chinese People's Healt. 2007; 19(3): 219-220.

**17.** Liu G, Yang Y, Han J, Di H. Investigation of depression in nurses working in Grade-II Class-A hospitals. Journal of Nursing Administration. 2009; 9(6): 22-23.

**18.** Wang Y. An epidemiological survey on primary headache disorders among nursing staff in north China [Master]: Medical School of Chinese PLA; 2015.

**19.** Ren C, Tang W, Wang W. Investigation and analysis of mental health status of employed nurses. Guide of China Medicine. 2011; 9(17): 168-170.

**20.** Cheng r, Guirong S. Investigation and Analysis of the depressive mood of 247 nurses in Dalian. Journal of Nursing Administration. 2004; 4(11): 12-14.

**21.** Yuan Y, Wang B, Wang X, Jin K. Survey on Mental Health Status of the Medical Staff. Nursing Journal of Chinese People's Liberation Army. 2007; 24(12A): 22-23;44.

**22.** Wang L, Luo S, Wu Q, Yang Y, Zhang J. Effect of Work Stress on Emotion of Military Nurses. Nursing Journal Of Chinese Peoples Liberation Army. 1996; 13(1): 1-4.

**23.** Tu S, Lin N, Xie L, Zou Q. Investigation and analysis of the mental health of the nurses in the basic hospital. Inner Mongol Journal of Traditional Chinese Medicine. 2013; 32(8): 75-76.

**24.** Huang L, Wang C, Fan L, Jiang Z, Jiang W. Analysis on the correlation between the job stress sources and the emotional state in nurses of hemodialysis rooms. International Journal of Nursing. 2013; 32(8): 1647-1649.

**25.** Zhu H. Comparison of Psychological Health Status Between Nurses at Materni ty and Nurses at General Surgery Ward. INNER MONGOLIA MEDICAL JOURNAL. 2009; 41(4): 504-506.

**26.** Qu L. Analysis of depressive symptoms in self-assessment of nurses. Health Psychology Journal. 1999; 7(4): 475-477.

**27.** Hong R, Huang H, Ying W, Xiao H, Wang M, Wang Y. Mental Health and Correlative Analysis of Nurses in Operating Room. Modern nurse. 2003; 9(9): 676-677.

**28.** Huang H, Xian M, HuiyingTan, Wang M, Wang Y, Cheng Y. Study on the Status of Depression of Clinical Nurses in Tumor Sections and Its Correlative Factors. Modern Clinical Nursing. 2004; 3(1): 1-3.

**29.** Xiang L, He C, Fu L. The correlation between acquired wisdom level and anxiety, depression in psychiatric nurses. Henan Medical Research. 2016; 25(6): 987-988.

**30.** Zhang H, Liu R, Chen S. A study on the psychological stress of young nurses in the face of death and its countermeasures. Journal of Qiqihar Medical College. 2009; 30(15): 1926-1927.

**31.** Deng X, Zhu Ze, Liao Y. Investigation and analysis of the anxiety and depression status of 96 cases of psychiatric nurses. China Medical Herald. 2011; 8(5): 122-123.

**32.** Lin C, Tang Q, Le S. Investigation and analysis of mental health status and its influencing factors of nurses in emergency department. Chinese General Practice Nursing. 2010; 8(6A): 1482-1484.

**33.** Gong Y, Han T, Yin X, Yang G, Zhuang R, Chen Y, et al. Prevalence of depressive symptoms and work-related risk factors among nurses in public hospitals in southern China: a cross-sectional study. Sci Rep. 2014; 4: 7109. https://doi.org/10.1038/srep07109 PMID: 25427988.

**34.** Zhu L. Causes and Countermeasures of work stress in NICU Nurses. China Health Industry. 2011; 8(7): 65.

**35.** Pan S, Zhou Y, Lin W, Hong Z, Xie H, Lin J, et al. Relationship between depression status and trait coping style of nurses and their influencing factors in a professional psychiatric hospitals in Guangzhou city. Chinese Journal of Modern Nursing. 2013; 19(23): 2745-2747.

**36.** Li N, Yao Y. Correlation and influence factors of coping style and mental disorders:a study in nurses from a medical center. Anhui Medical Journal. 2016; 37(6): 764-767.

**37.** Zhong M, Qin J, Guo X. Investigation and analysis of psychological state of nurses in outpatient and emergency department. Today Nurse. 2013; (12): 99-100.

**38.** Gu F. Investigation and Analysis on anxiety and Depression and Social support of Psychiatric Nursing staff. Shanghai Nursing Journal. 2006; 6(4): 17-19.

**39.** Wang J, Xu C, Wang Y. An Analysis of the Anxiety, Depression and Pressure of NICU Nurses and the Coping Strategy. Journal of Nursing. 2009; 16(2B): 68-69.

**40.** Zheng J, Jiang X, Huang X. Investigation on depressive mood of Nurses in a Maternal and Child Hospital. Chinese Journal of Industrial Hygiene and Occupational Diseases. 2018; 36(8): 618-621. https://doi.org/10.3760/cma.j.issn.1001-9391.2018.08.014 PMID: 30317815.

**41.** Jia Z, Li H. A study on the association of depression with presenteeism in clinical nurses in a 3A grade hospital in Zhengzhou. Chongqing Medicine. 2015; 44(20): 2874-2875.

**42.** Chen C. Correlation analysis of anxiety, depression and social support of nurses in enterprise restructuring hospitals. China Practical Medical. 2014; 9(16): 250-252.

**43.** Song D. Associations of anxiety and depression with social support in contract nurses Seek Medical and Ask the Medicine. 2012; 10(03): 82.

**44.** Luo Y, Cai T. The Relationship among Anxious, Depressive Mood and Family Function of Nurses. China Journal of Health Psychology. 2011; 19(12): 1458-1459.

**45.** Liu Y. Investigation and Analysis on the present situation of Depression in Nursing population in Xining City. Modern Medicine & Health. 2010; 26(13): 1999-2000.

**46.** Yan F, Quan H, Zhang R. An investigation on depression emotions of nurses in operating room. Chinese Nursing Research. 2004; 18(3B): 482-483.

**47.** Hui C. Anxiety, depression status and correlation analysis of the quality life among community hospital nurses [Master]: Jinzhou Medical University; 2016.

**48.** Fang H, Zhao X, Yang H, Sun P, Li Y, Jiang K, et al. Depressive symptoms and workplace violence-related risk factors among otorhinolaryngology nurses and physicians in Northern China: a cross-sectional study. BMJ Open. 2018; 8(1). PMID: WOS:000431743500039.

**49.** Liu J, Bao L. Investigation and Analysis of depressive mood of Psychiatric Nurses and its Countermeasures. Hebei Medical Journal. 2006; 28(8): 778-779.

**50.** Shi R, Li D, Xia J, Pei Q, Zhou Y, Li C, et al. Mental health status of nursing staff in the three-level hospitals in Tangshan city. Shanxi Medical Journal. 2015; 44(21): 2467-2469.

**51.** Wei Y, Fu H, Shen X. Investigation and study on depressive state of nurses. Health Vocational Education. 2007; 25(16): 95-96.

**52.** Wang Z. Level of job burnout and psychological factors related to it in medical workers [Master]: Xinxiang Medical University; 2015.

**53.** Liu X, Lu S, Dong B, Fang H. Suicidal ideation and its related factors among nurses of oncology departments of four cities in China. China Journal of Modern Medicine. 2018; 28(08): 76-81.

**54.** Sun L, Jianahemaiti•Hafula, Yuan Y, Ren J. Influence of doctor-patient relationship attitude on anxiety and depression of military hospital nurses. OCCUPATION AND HEALTH. 2019; 35(2): 153-155,159.

**55.** Wang D, Hua M, Pan Y, Bai Y, Han D. Analysis on status and influencing factors of depression among nurses in a 3A grade hospital in Beijing. Chinese Journal of Health Statistics. 2018; 35(2): 225-227.

**56.** Cheng J, Zhang Y, Han S, Chu Y. Investigation on Depressive Mood of Clinical Nurses. Chinese Nursing Research. 2002; 16(3): 138-139.

**57.** Jiang P, Su X, Jia M. A study on correlation between depression and social support in military nurses in Beijing. Chinese Journal of Modern Nursing. 2013; 19(19): 2240-2243.

**58.** Huang L, Yang R. Analysis of Depression of Nurses. China Health Industry. 2015; 12(5): 171-172.

**59.** Liu Y, Zhang J, Fu X. Research on relationships among job burnout, depression and social support of tumor hospital nurses. Journal of Nursing Administration. 2014; 14(5): 331-333.

**60.** Liu Z, Zhong M, Hai Y, Du Q, Wang A, Xie D. Influencing factors on depression among medical staff in Hunan province under ordinal regression analysis. Chinese Journal of Epidemiology. 2012; 33(11): 1115-1118. PMID: 23290893.

**61.** Ouyang N. A study on the mental heathy status of 17170 medical staff and its determinate factors [Master]: Central South University; 2012.

**62.** Gong Y, Zhong W. Correlation study of anxiety-depression emotion and job stress among psychiatric male nurses. Journal of Clinical Psychosomatic Diseases. 2012; 18(1): 69-70.

**63.** Han X, Li H, Chen W, Liu M. Research on relationships among job stress, social support, coping style and anxiety-depression emotion of nurses. Journal of Neuroscience and Mental Health. 2013; 13(6): 632-635.

**64.** Liang Q. The Assessment of Depression in Psychiatric Nurses and the Analysis of Influential Factors. Chinese Journal of Nursing. 1998; 33(6): 321-323.

**65.** Zhao H. Study on depressive state and work stress of nurses in emergency department. Chinese Journal of Nursing. 2007; 4(4): 35-36.

**66.** Xu Q. Influence of working pressure on the depression and anxiety of psychiatric nurses. Journal of Qilu Nursing. 2011; 17(21): 13-14.

**67.** Xu Q, Xu M, Qiao Y, Liu Z. The relation of anxiety and depression with job stress among psychiatric nurses. Journal of Psychiatry. 2010; 23(4): 250-252.

**68.** Zhou C, Wang Z, Li X, Han Q, Wang B. Investigation and Analysis of Depression in Clinical and Psychiatry Nurses. Medical Journal of Chinese People Health. 2004; 16(9): 588-589.

**69.** Mo X, Yu Y, Wang W, He W. Influence of rumination on depression level of nurses. Journal of nursing and rehabilitation. 2019; 18(5): 35-38. https://doi.org/10.3969/j.issn.1671-9875.2019.05.009.

**70.** Chen C, Zhong B, Hua Y, Nie F, Wang X. A study on the detection rate of depression and anxiety, co-illness and related factors of nurses in grade 3a general hospital. Journal of Nursing. 2013; 20(5): 63-66.

**71.** Ding Y, Wang Y. A survey of quality of sleep in clinical nur ses of the Class A ter tiary hospital. Beijing Medical Journal. 2007; 29(11): 667-670.

**72.** Wang Y, Liu G, Zhou X, Sheng P, Cui F, Shi T. Mediating effect of mental elasticity on occupational stress and depression in female nurses. Chinese Journal of Industrial Hygiene and Occupational Diseases. 2017; 35(6): 436-439.

**73.** Zhou L, Zhou L, Lan X. Investigation and analysis of anxiety and depression in the employment of nurses. Today Nurse. 2006; (01): 99-101.

**74.** Zhang X, Wu L, Li S. An analysis of nursing staff's negative mood and relative factors. Tianjin Journal of Nursing. 2006; 14(2): 63-65.

**75.** Li J. An investigation on status of physical and mental health of paediatric nurses in Zaozhuang City and the corresponding countermeasures. Journal of Qilu Nursing. 2016; 22(13): 59-60.

**76.** Zhang J, Wang A, Zhang S, Lu Q. Study of the nurses' job stressor, psychological health and coping styles in ICU. Journal of Nursing Administration. 2004; 4(8): 1-4.

**77.** Yu R, Liu H, Xie J, Sun Y, He Y. Investigat ion and Analysis of Mental Health Status of Clinical Staff in General Hospital. China Journal of Health Psychology. 2010; 18(2): 155-157.

**78.** Jiao Y, Huang J, Sun L, Tang G, Pan Y, He X. Investigative Study of the Job Stressors and Mental Health of Obstetrical Nurses. International Journal of Nursing. 2006; 25(12): 978-980.

**79.** Geng X. Analysis of anxiety and depression in clinical first-line nurses and their spouses. Chinese Journal of Modern Nursing. 2012; 18(30): 3657-3658.

**80.** Gao Y, Pan B, Wu H, Wang L. A Survey Study on Depressive Symptoms of Nurses and Their Correlation with Nurse-patient Relationship. Journal of China Medical University. 2011; 40(6): 547-548,552.

**81.** Gao YQ, Pan BC, Sun W, Wu H, Wang JN, Wang L. Depressive symptoms among Chinese nurses: prevalence and the associated factors. J Adv Nurs. 2011; 68(5): 1166-1175. Epub 2011/09/29. https://doi.org/10.1111/j.1365-2648.2011.05832.x PMID: 21950775.

**82.** Zhao X, Gao F, Liu L, Wu H. Prevalence and associated factors of depressive symptoms among the female nurses in liaoning province. Journal of China Medical University. 2012; 41(7): 667-670.

**83.** Wu H, Wang J, Yang X, Wang Y, Wang L. Prevalence and associated factors of depressive symptoms among hospital staff. China Preventive Medicine. 2010; 11(9): 902-906.

**84.** Wu H, Hu B, Wang J, Chi T, Wang L. Relationship between depressive symptom and occupational stress among nurse. Chinese Journal of Public Health. 2010; 26(5): 595-597.

**85.** Wang B, Chen Z, Liu G, Hong H, Li R. Effect of marital status on climacteric depression in clinical nurses. Chinese Journal of Gerontology. 2012; 32(20): 4482-4483. https://doi.org/10.3969/j.issn.1005-9202.2012.20.057.

**86.** Yuan J, Shang C, Zhang M, Guo X, Pang J, Wang J. Influencing factors of anxiety and depression among medical staff and its relationship with psychiatric resilience in 4 hospitals of Tangshan City in 2016. Occupation and Health. 2017; 33(21): 2918-2922.

**87.** Li X, Guo Y, Lu W, Wang S, Chen K. Association between social psychological factors and depressive symptoms among healthcare workers. Chinese Journal of Industrial Hygiene and Occupational Diseases. 2006; 24(8): 454-457.

**88.** Wan X. Investigation on the situation of female nurses'injury and depressive symptoms in Jiujiang public hospital hospital. Chinese General Practice Nursing. 2019; 17(32): 4079-4081. https://doi.org/10.12104/j.issn.1674-4748.2019.32.032.

**89.** Yan L, Guo X, Pang J, Zhao W, Peng Y, Ying L, et al. Study on clinical heterogeneity of depressive symptoms in medical staff in Tangshan City. OCCUPATION AND HEALTH. 2019; 35(17): 2358-2361.

**90.** Huang X, Li F, Wang Z. Depression among the female medical staff in general hospitals. Medical Journal of Chinese People's Health. 2009; 21(17): 2078-2079.

**91.** Zhang L, Quan Y, Zhao M. Investigation on the status of low back pain in clinical nurses and analysis of its influencing factors Chinese General Practice Nursing. 2017; 15(19): 2311-2313.

**92.** Li X, Hou Y, Zhang L. Investigation and Analysis on Depression of Young Nurses. Journal of Nursing Science. 2004; 19(9): 54-55.

**93.** Dai C. Investigation of depression in male nursing workers. Changzhou Practical Medicine. 2014; 30(6): 397-398.

**94.** Zhou Y. Investigation of Social Support and Depression and Feeling on Work of Nurses in Emergency Department. Today Nurse. 2004; (6): 5-7.

**95.** Li Y, Zhang X, Wang M. The correlation of serum concentration of copper and prevalence of depressive mood in psychiatric night shift nurses. Chinese Journal of Practical Nursing. 2016; 32(2): 131-135.

**96.** Li Y, Feng Y, Wang L, Fan J. Surveys of anxiety-depression status in psychiatric shift nurses. Journal of Clinical Psychosomatic Diseases. 2016; 22(6): 64-65.

**97.** Wang X, Xu J, Wang C, Lu P. Thoughts on the Hope level and emotional State of retired nurses in the Eastern Coastal areas of Zhejiang Province Journal of Traditonal Chinese Medicine Management. 2018; 26(13): 16-19.

**98.** Zhang X, Tang F, Xia Y. Evaluation on the effect of peer education on depression for nurses in blood purification center. Journal of Preventive Medicine. 2017; 29(9): 961-963.

**99.** Ke C, Xu Y. A study on the correlation between depressive symptoms and occupational stress in ICU nurses and the corresponding countermeasures. Journal of Military Surgeon in in Southwest China. 2015; 17(4): 464-466.

**100.** He H, Yin S, Li W. Investigation on the psychological status of head nurses under the mode of post management and the corresponding countermeasures. Journal of Qilu Nursing. 2017; 23(6): 31-32.

**101.** Wang X. A study on anxiety and depression psychology of nurses in emergency department. China Modern Medicine. 2011; 18(19): 118-119.

**102.** Dai C, Qiu H, Huang Q, Hu P, Hong X, Tu J, et al. The effect of night shift on sleep quality and depressive symptoms among Chinese nurses. Neuropsychiatric Disease and Treatment. 2019; 15: 435-440. https://doi.org/10.2147/ndt.s190689.
