# Supplementary material for: Prevalence of depressive symptoms among nurses in China: A systematic review and meta-analysis
Source: PLoS One. 2020 Jul 7;15(7):e0235448. doi: 10.1371/journal.pone.0235448 (PMC7340293; doi:10.1371/journal.pone.0235448)
Supplement: S2 Table — (DOCX) [file pone.0235448.s002.docx]

Table S2. Quality evaluation of the 101 studies included in the meta-analysis

| Study code | ① | ② | ③ | ④ | ⑤ | ⑥ | ⑦ | ⑧ | ⑨ | ⑩ | ⑪ | Score of Quality |
| --- | --- | --- | --- | --- | --- | --- | --- | --- | --- | --- | --- | --- |
| 1 | Y | N | Y | - | Y | N | N | Y | N | Y | - | 5 |
| 2 | Y | N | N | - | U | N | N | Y | N | Y | - | 3 |
| 3 | Y | N | Y | - | U | N | N | N | N | Y | - | 3 |
| 4 | Y | N | N | - | U | N | N | N | N | Y | - | 2 |
| 5 | Y | N | N | - | Y | N | Y | Y | N | Y | - | 5 |
| 6 | Y | N | N | - | U | N | N | Y | N | N | - | 2 |
| 7 | Y | N | Y | - | Y | Y | N | N | N | Y | - | 5 |
| 8 | Y | N | N | - | Y | N | N | Y | N | Y | - | 4 |
| 9 | Y | Y | Y | - | Y | N | - | Y | - | Y | - | 6 |
| 10 | Y | N | N | - | U | N | N | N | N | Y | - | 2 |
| 11 | Y | N | N | - | U | N | - | N | - | Y | - | 2 |
| 12 | Y | N | N | - | U | N | N | Y | N | Y | - | 3 |
| 13 | Y | N | N | - | Y | N | - | N | - | Y | - | 3 |
| 14 | Y | Y | Y | - | Y | N | N | N | N | Y | - | 5 |
| 15 | Y | N | N | - | U | N | N | N | N | Y | - | 2 |
| 16 | Y | N | N | - | U | N | N | N | N | Y | - | 2 |
| 17 | Y | Y | Y | - | U | N | N | Y | N | Y | - | 5 |
| 18 | Y | Y | Y | - | U | N | Y | Y | N | Y | - | 6 |
| 19 | Y | Y | N | - | U | Y | N | N | N | Y | - | 4 |
| 20 | Y | N | N | - | U | N | N | Y | N | Y | - | 3 |
| 21 | Y | N | Y | - | U | Y | N | N | N | Y | - | 4 |
| 22 | Y | N | N | - | U | N | N | Y | N | Y | - | 3 |
| 23 | Y | N | N | - | U | N | - | Y | - | Y | - | 3 |
| 24 | Y | N | Y | - | U | N | - | N | - | Y | - | 3 |
| 25 | Y | N | N | - | U | N | N | N | N | Y | - | 2 |
| 26 | Y | N | N | - | U | N | N | Y | N | Y | - | 3 |
| 27 | Y | N | N | - | U | N | N | Y | N | Y | - | 3 |
| 28 | Y | Y | N | - | U | N | Y | Y | N | Y | - | 5 |
| 29 | Y | Y | N | - | U | N | - | N | - | Y | - | 3 |
| 30 | Y | Y | N | - | U | N | - | N | - | Y | - | 3 |
| 31 | Y | Y | N | - | Y | N | - | Y | - | Y | - | 5 |
| 32 | Y | N | N | - | Y | N | - | Y | - | Y | - | 4 |
| 33 | Y | Y | Y | - | Y | N | Y | N | N | Y | - | 6 |
| 34 | Y | N | N | - | U | N | - | N | - | Y | - | 2 |
| 35 | Y | Y | N | - | Y | N | N | Y | N | Y | - | 5 |
| 36 | Y | N | Y | - | Y | Y | N | Y | N | Y | - | 6 |
| 37 | Y | N | Y | - | Y | N | - | Y | - | Y | - | 5 |
| 38 | Y | Y | N | - | Y | N | - | Y | - | Y | - | 5 |
| 39 | Y | N | Y | - | U | N | - | Y | - | Y | - | 4 |
| 40 | Y | Y | Y | - | Y | Y | N | Y | N | Y | - | 7 |
| 41 | Y | Y | Y | - | U | Y | Y | Y | N | Y | - | 7 |
| 42 | Y | Y | Y | - | Y | N | N | Y | N | Y | - | 6 |
| 43 | Y | Y | Y | - | U | N | - | Y | - | Y | - | 5 |
| 44 | Y | N | N | - | U | N | N | Y | N | Y | - | 3 |
| 45 | Y | N | Y | - | U | N | N | N | N | Y | - | 3 |
| 46 | Y | N | Y | - | U | N | N | N | N | Y | - | 3 |
| 47 | Y | Y | Y | - | Y | Y | Y | Y | N | Y | - | 8 |
| 48 | Y | N | Y | - | U | N | N | Y | N | Y | - | 4 |
| 49 | Y | Y | N | - | U | N | N | N | N | Y | - | 3 |
| 50 | Y | Y | N | - | U | N | N | N | N | Y | - | 3 |
| 51 | Y | N | N | - | Y | N | - | Y | - | Y | - | 4 |
| 52 | Y | Y | Y | - | Y | N | N | Y | N | Y | - | 6 |
| 53 | Y | Y | Y | - | Y | N | N | Y | N | Y | - | 6 |
| 54 | Y | Y | Y | - | Y | N | N | Y | N | Y | - | 6 |
| 55 | Y | N | N | - | U | N | Y | Y | N | Y | - | 4 |
| 56 | Y | N | N | - | U | N | N | Y | N | Y | - | 3 |
| 57 | Y | Y | Y | - | Y | N | Y | Y | N | Y | - | 7 |
| 58 | Y | N | N | - | U | N | - | Y | - | Y | - | 3 |
| 59 | Y | Y | Y | - | Y | N | N | Y | N | Y | - | 6 |
| 60 | Y | Y | N | - | U | Y | N | Y | N | Y | - | 5 |
| 61 | Y | Y | Y | - | Y | Y | Y | Y | N | Y | - | 8 |
| 62 | Y | Y | N | - | U | N | - | N | - | Y | - | 3 |
| 63 | Y | N | N | - | U | N | N | N | N | Y | - | 2 |
| 64 | Y | N | N | - | Y | N | N | Y | N | Y | - | 4 |
| 65 | Y | N | N | - | Y | N | N | Y | N | Y | - | 4 |
| 66 | Y | Y | N | - | U | N | - | N | - | Y | - | 3 |
| 67 | Y | Y | N | - | U | N | N | N | N | Y | - | 3 |
| 68 | Y | N | Y | - | Y | N | N | Y | N | Y | - | 5 |
| 69 | Y | Y | Y | - | U | N | N | Y | N | Y | - | 5 |
| 70 | Y | N | Y | - | U | N | N | Y | N | Y | - | 4 |
| 71 | Y | N | Y | - | U | N | Y | Y | N | Y | - | 5 |
| 72 | Y | Y | Y | - | U | N | N | Y | N | Y | - | 5 |
| 73 | Y | N | N | - | Y | N | - | Y | - | Y | - | 4 |
| 74 | Y | N | N | - | Y | N | N | Y | N | Y | - | 4 |
| 75 | Y | Y | Y | - | Y | N | N | Y | N | Y | - | 6 |
| 76 | Y | N | Y | - | U | N | N | N | N | Y | - | 3 |
| 77 | Y | N | N | - | Y | N | N | Y | N | Y | - | 4 |
| 78 | Y | N | N | - | U | N | N | N | N | Y | - | 2 |
| 79 | Y | Y | Y | - | U | N | N | Y | N | Y | - | 5 |
| 80 | Y | N | Y | - | Y | N | N | N | N | Y | - | 4 |
| 81 | Y | Y | Y | - | N | N | N | Y | N | Y | - | 5 |
| 82 | Y | Y | Y | - | U | N | N | Y | N | Y | - | 5 |
| 83 | Y | Y | Y | - | U | N | N | Y | N | Y | - | 5 |
| 84 | Y | Y | Y | - | U | N | N | Y | N | Y | - | 5 |
| 85 | Y | Y | N | - | U | N | N | Y | N | Y | - | 4 |
| 86 | Y | N | Y | - | Y | N | N | Y | N | Y | - | 5 |
| 87 | Y | N | Y | - | U | N | N | Y | N | Y | - | 4 |
| 88 | Y | Y | Y | - | U | Y | Y | Y | N | Y | - | 7 |
| 89 | Y | Y | Y | - | U | N | N | Y | N | Y | - | 5 |
| 90 | Y | Y | N | - | U | N | N | N | N | N | - | 2 |
| 91 | Y | Y | Y | - | U | N | N | Y | N | Y | - | 5 |
| 92 | Y | Y | N | - | Y | N | N | Y | N | Y | - | 5 |
| 93 | Y | Y | Y | - | U | N | N | N | N | Y | - | 4 |
| 94 | Y | Y | N | - | U | N | N | N | N | Y | - | 3 |
| 95 | Y | Y | Y | - | U | N | - | N | - | Y | - | 4 |
| 96 | Y | Y | Y | - | U | N | N | N | N | Y | - | 4 |
| 97 | Y | Y | Y | - | U | N | - | Y | - | Y | - | 5 |
| 98 | Y | Y | Y | - | Y | N | N | Y | N | Y | - | 6 |
| 99 | Y | Y | N | - | U | N | - | N | - | Y | - | 3 |
| 100 | Y | Y | N | - | Y | N | - | Y | - | Y | - | 5 |
| 101 | Y | Y | Y | - | U | N | - | N | - | Y | - | 4 |
| 102 | Y | N | Y | - | Y | N | Y | Y | - | Y | - | 6 |

Y, yes; N, no; U, unclear; Items 4 and 11 were not appropriate for the studies included in the meta-analysis. An item would be scored ‘0’ if it was answered ‘NO’ or ‘UNCLEAR’; if it was answered ‘YES’, then the item scored ‘1’. Article quality was assessed as follows: low quality = 0–3; moderate quality = 4–7; high quality = 8–11.
