# Supplementary material for: Prevalence of depressive symptoms among nurses in China: A systematic review and meta-analysis
Source: PLoS One. 2020 Jul 7;15(7):e0235448. doi: 10.1371/journal.pone.0235448 (PMC7340293; doi:10.1371/journal.pone.0235448)
Supplement: S1 Appendix — (DOC) [file pone.0235448.s005.doc]

The search string for our study: ((((((Chinese) OR China)) AND ((Nurse) OR Nurses)) AND (((((Depression) OR Mental Health Disorder) OR Major Depression Disorder) OR mood disorder) OR affective disorder)) AND (((Prevalence) OR Frequency) OR Epidemiology))

Search strategy in PubMed/MEDLINE

#1 Prevalence

#2 Frequency

#3 Epidemiology

#4 #1 OR #2 OR #3

#5 Depression

#6 Mental Health Disorder

#7 Major Depression Disorder

#8 Mood disorder

#9 Affective disorder

#10 #5 OR #6 OR #7 OR #8 OR #9

#11 Nurses

#12 Nurse

#13 #11 OR #12

#14 China

#15 Chinese

#16 #14 OR #15

#17 #4 AND #10 AND #13 AND #16
